# Tools for assessing implementation of health promotion standards in hospital/health service settings

**DOI:** 10.1093/eurpub/ckac131.323

**Published:** 2022-10-25

**Authors:** K Katsaros, O Groene

**Affiliations:** 1 International Secretariat, International HPH Network, Hamburg, Germany; 2 Research & Innovation, OptiMedis AG, Hamburg, Germany

## Abstract

The International Network of Health Promoting Hospitals and Health Services (HPH) was initiated by the World Health Organization as a settings approach toward reorienting health services. Over 600 members from 30 countries promote the integration of health promotion into the hospital/ health service setting. The International HPH Network developed the 2020 Standards for Health Promoting Hospitals and Health Services, representing years of experience and expertise reflected in 5 standards, 18 substandards, and 85 standard statements. Complementary self-assessment tools were developed to operationalize the standards and to identify concrete measurable elements against which each standard can be measured. The aim of the tools is to assist hospitals and health services in transforming the setting into a health promoting one. The standards are comprehensive and address topics to encourage the complete refocusing of an organization’s strategy and to stimulate a process of continuous internal improvement. Standards address management, patient-centered care, occupational health, health literate organizations, environmental sustainability, and target groups such as the elderly and children and adolescents. Excel and pdf tools may be utilized by institutions to measure and track progress in implementing each of the standards. The tools state measurable elements for each standard that were identified by an expert panel of HPH members and external subject experts to be directly observable and applicable across various regional and institutional contexts. As part of internal and external assessment processes, standards can be rated on a scale from 1 (not implemented) to 10 (fully implemented). The tools store data and generate graphs that allow organizations to assess their level of performance, identify areas for improvement, and devise data-driven action plans.

**Key messages:**

• Progress towards transforming the setting must be tracked and included in an organization’s continuous internal improvement processes.

• The transformation of the hospitals/health service setting into a health promoting environment results in better health outcomes for patients, families, the community, and the environment.

